# Potassium 4‐Methoxysalicylate (4MSK) Exerts a Skin Lightening Effect by Acting on Melanocytes and Keratinocytes

**DOI:** 10.1111/jocd.70112

**Published:** 2025-03-12

**Authors:** Yutaka Shirasugi, Takako Shibata, Saaya Koike, Masao Hara, Kiyotaka Hasegawa, Masato Iino, Aska Sonoki, Yoko Funasaka

**Affiliations:** ^1^ MIRAI Technology Institute, Shiseido Co. Ltd. Yokohama Japan; ^2^ Department of Dermatology Ikebukuro West Gate Hospital Tokyo Japan

**Keywords:** hyperpigmentation, keratinization, melanogenesis, potassium 4‐methoxysalicylate, skin lightening

## Abstract

**Background:**

Hyperpigmentation is a common acquired disorder that can be a cosmetic concern for many individuals. To reduce and prevent hyperpigmentation, numerous skin lightening agents have been developed. Potassium 4‐methoxysalicylate (4MSK) is a skin lightening agent that was approved as an active skin lightening ingredient of quasi‐drugs by the Ministry of Health, Labour and Welfare of Japan in 2003. For over 20 years, 4MSK has been widely used in quasi‐drug and cosmetic products. However, the mechanism of action and efficacy of 4MSK on skin pigmentation and skin lightness have not been publicly reported.

**Aims:**

This study aimed to assess the mechanism of action and the efficacy of 4MSK on facial pigmentation.

**Methods:**

The mechanism of action of 4MSK on epidermal cells was investigated using human melanocytes, human keratinocytes, and human 3D epidermal equivalents. The efficacy of 4MSK on facial pigmentation was evaluated by a human clinical study, which is a double‐blind, split‐face, placebo‐controlled, paired‐design study.

**Results:**

4MSK significantly suppressed melanin content in cultured human melanocytes and a 3D epidermal equivalent. It also promoted gene expression of differentiation markers of human keratinocytes. In the clinical study, a 4MSK formulation significantly increased skin lightness values in both pigmented and non‐pigmented areas of cheek skin. In addition, it reduced the desquamation area ratio of the cheek.

**Conclusions:**

4MSK reduces skin pigmentation and contributes to brighter skin by acting on both melanocytes and keratinocytes.

## 1 Introduction

Exposure to ultraviolet rays causes skin pigmentation such as solar lentigo and melasma. Because skin pigmentation is a major skin concern, especially for individuals with fair skin tones, the Asian market for skin lightening agents has been expanding [1]. Therefore, numerous chemical compounds have been developed as skin lightening agents. The most common skin lightening agents include hydroquinone, arbutin, and kojic acid, but their use is restricted in some countries [2, 3]. In Japan, skin lightening cosmetics are classified as quasi‐drugs, and approximately 20 skin lightening active ingredients are approved by the Japanese administrative authority [3, 4]. The mechanism of action of these active ingredients can be broadly classified into two groups: those that directly or indirectly suppress melanin production in melanocytes and those that promote proliferation of keratinocytes and accelerate epidermal turnover to suppress melanin accumulation [3, 4]. Potassium 4‐methoxysalicylate (4MSK), a derivative of salicylic acid (SA) that was approved in 2003 as a skin lightening active ingredient, suppresses melanin production by inhibiting tyrosinase, a pivotal enzyme involved in the melanogenesis process [3, 4]. Although 4MSK has been used commercially as a skin lightening agent for over 20 years, its efficacy and mechanism of action for skin pigmentation have not been publicly reported in scientific journals.

In this study, we first investigated the effects of 4MSK on normal human melanocytes and keratinocytes in vitro. Next, we investigated the skin lightening effect of a formulation containing 3% 4MSK on facial skin pigmentation with a human clinical study. In addition, the effect of 4MSK on skin surfaces was investigated by collecting the facial stratum corneum.

## 2 Materials and Methods

### 2.1 Measurement of Melanin Content in Cultured Human Melanocytes

Melanin contents of normal human epidermal melanocytes‐highly pigmented (NHEM‐HP) cells derived from African American neonatal foreskin (Kurabo Industries Ltd., Osaka, Japan) were measured as previously described with minor modifications [5]. NHEM‐HP cells were seeded in 12‐well plates and cultured for 2 days in DermaLife basal medium (Kurabo Industries Ltd.) supplemented with 6 mM L‐glutamine, 1 μM epinephrine, 5 μg/mL insulin, 1% StiMel 8 LifeFactors (human epidermal growth factor (hEGF), bovine pituitary extract (BPE), hydrocortisone, transferrin, fetal bovine serum, human fibroblastic growth factor‐B, endothelin), 50 μg/mL ascorbic acid, 0.2 mM CaCl_2_, 30 μg/mL gentamicin, and 15 ng/mL amphotericin B. Next, they were treated with α melanocyte‐stimulating hormone (0.3 μM; Sigma‐Aldrich, St. Louis, MO, USA) and varying concentrations of 4MSK for 14 days. The medium was replaced every 2 or 3 days. Cell viability was determined using alamarBlue (Thermo Fisher Scientific, Waltham, MA, USA) according to the manufacturer's protocol. Cells were then washed with Hank's Balanced Salt Solution and lysed in cell lysis buffer containing 1 N NaOH for 2 h at 80°C. The absorbance of the resulting lysate at 405 nm was measured to determine the total melanin content.

### 2.2 Measurement of Melanin Contents in a 3D Epidermal Equivalent

A human three‐dimensional (3D) epidermal equivalent model reconstructed from Asian epidermal cells (MelanoDerm MEL‐300‐A; MatTek, Ashland, MA, USA) was treated with or without a 0.05% 4MSK aqueous solution for 14 days in EPI‐100NMM113 medium containing bFGF, α‐MSH, and KGF. The maintenance medium and 4MSK solution were replaced every 2 days. To measure melanin contents, 3D skin models were collected, sonicated, and then centrifuged (4°C, 2300 × *g*, 5 min). NaOH (200 μL, 0.2 N) was added to the obtained cell aggregates, and the mixture was boiled at 80°C for 30 min to extract intracellular melanin. The absorbance at 405 nm of the obtained supernatant was measured and defined as the amount of melanin contained in the cells. Amounts of melanin were quantified using a calibration curve obtained with synthetic melanin (Sigma‐Aldrich) and then standardized by the amount of protein. Skin samples were fixed in a 4%‐paraformaldehyde phosphate‐buffer solution (Nacalai Tesque, Kyoto, Japan) overnight, embedded in paraffin by the conventional method, and cut into 5‐μm thick sections with a microtome (SM2000R, Leica Biosystems, Nussloch, Germany). Paraffin sections were subjected to Fontana–Masson staining for melanin detection.

### 2.3 Ex Vivo Human Skin Explants

NativeSkin models were purchased from Genoskin (Toulouse, France). These models are ex vivo punch biopsies obtained from unexposed areas of 30‐year‐old Caucasian female donors without skin disease. Informed consent from donors and ethical approval were obtained for the commercialization and experimental use of the skin biopsies. NativeSkin models were incubated in a CO_2_ incubator at 37°C. After 24 h, 1% 4MSK or dH_2_O was added to the top of the skin sample, which was further incubated for 24 h. After repeating this agent addition for 4 days, skin samples were collected.

### 2.4 Tyrosinase Inhibition Assay

The inhibitory effect of 4MSK on mushroom tyrosinase was determined as described in a previous study with some modifications [6]. Briefly, 140 μL of 4MSK solution in 0.1 M phosphate buffer was mixed with 20 μL of mushroom tyrosinase (500 U/mL, Sigma‐Aldrich). The mixture was incubated at 37°C for 5 min and then added to 40 μL of L‐DOPA (4 mM, Nacalai Tesque). After incubating for 5 min at 37°C, the absorbance at 475 nm of the reaction mixture was measured. The inhibition rate of L‐DOPA oxidation was calculated as follows: % inhibition = 100 − (*B*/*A* × 100), where *A* = ΔOD_475_ in 5 min without sample, and *B* = ΔOD_475_ in 5 min with tested sample.

### 2.5 Cell Culture of Human Keratinocytes and Quantitative RT‐PCR Analysis

Normal human epidermal keratinocytes (NHEK, Kurabo Industries Ltd.) derived from Caucasian neonatal foreskin were seeded in 24‐well plates and cultured overnight in HuMedia‐KG2 medium (Kurabo Industries Ltd.) containing 10 μg/mL Insulin, 0.1 ng/mL hEGF, 0.67 μg/mL Hydrocortisone, 50 μg/mL gentamicin, 50 ng/mL amphotericin B, and 0.4% BPE. Subsequently, they were cultured with or without 0.8 mM 4MSK for 2 days. Next, total RNA was isolated from the cells using an RNeasy Mini Kit (Qiagen, Courtaboeuf, France). Subsequently, the first cDNA strand was synthesized from 1.5 μg of total RNA using a SuperScript VILO cDNA Synthesis Kit (Thermo Fisher Scientific). After the reaction, a quantitative polymerase chain reaction (qPCR) assay was performed using a Platinum SYBR Green qPCR SuperMix‐UDG Kit (Thermo Fisher Scientific) according to the manufacturer's protocol. Levels of RNA encoding the genes of interest were standardized using the transcript encoding the *RPL13A* gene in each sample. The sequences of primers are shown in Table S1.

### 2.6 Immunofluorescence Analysis of Human 3D Epidermal Equivalents

A human epidermal 3D model (EpiDerm EPI‐200; MatTek) was treated with or without 0.05% 4MSK aqueous solution for 3 days in EPI‐100MM medium, which is serum‐free medium based on DMEM. Subsequently, skin samples were fixed overnight in 4%‐paraformaldehyde phosphate buffer solution, embedded in paraffin by the conventional method, and cut into 3‐μm thick sections with a microtome (SM2000R, Leica Biosystems). Antigen retrieval was performed using HistoVT One pH 7.0 (Nacalai Tesque) at 90°C for 20 min. Sections were then incubated with anti‐filaggrin (Santa Cruz Biotechnology, Dallas, TX, USA) and anti‐loricrin (BioLegend, San Diego, CA, USA) antibodies at 4°C overnight, followed by incubation with secondary antibodies (Alexa Fluor 488 or 594; Abcam, Cambridge, UK) for 1 h at room temperature. After nuclear staining with Hoechst 33342 (Thermo Fisher Scientific), slides were mounted with Fluoromount‐G Anti‐Fade (Southern Biotech, Birmingham, AL, USA). All samples were analyzed using an Olympus BX51 fluorescence microscope (Olympus, Tokyo, Japan).

### 2.7 Human Clinical Study

This human clinical study was sponsored by Shiseido Co., Ltd., Japan, and conducted by a contract research organization (Institut d'Expertise Clinique Korea, Korea) between January and April 2020. This study was approved by the ethics committees of both Shiseido Co., Ltd. (IRB number: B01620) and Institut d'Expertise (IRB number: IECK(2)‐IRB‐19K516452) and conducted under the Ethical Guidelines for Clinical Research and the Declaration of Helsinki (UMIN000029305). All participants received an adequate explanation of the clinical study and provided written informed consent. The study was a double‐blind, split‐face, placebo‐controlled, paired‐design study. Thirty‐one Korean women aged 36–50 years with facial hyperpigmentation were enrolled. Subjects applied a formulation containing 3% 4MSK to one side of the face and a formulation without 4MSK (placebo) to the other side twice daily for 12 weeks. Subjects used the test products and usual skin care products, and sunscreen during the study period, but were prohibited from using products containing skin whitening ingredients.

### 2.8 Measurement of Skin Lightness Values

Skin lightness values of pigmented and non‐pigmented areas were measured using a spectrophotometer (CM‐2600d; Konica Minolta, Tokyo, Japan). Measurements were carried out three times to calculate the average L* (lightness) value. The VALUE parameter, which is an index that indicates the lightness of an image in the HSV color space, of a wide range of each cheek was calculated using photographs taken with the VISIA skin analysis imaging system (Canfield Scientific, Parsippany, NJ, USA) using an image analysis program Image Pro+ (Media Cybernetics, Rockville, MD, USA).

### 2.9 Measurement of Desquamation Areas of the Stratum Corneum

Skin corneocytes on the cheek were collected by two consecutive applications of tape stripping using black D‐SQUAME (Cuderm Corp, Dallas, TX, USA). Video images of samples were captured three times by Charm View (Moritex, Kanagawa, Japan) with a lens that magnifies 700× and processed with the aid of Image Pro+.

### 2.10 Statistical Analysis

A Welch's *t*‐test, Dunnett's test, or two‐sided Wilcoxon signed‐rank test was used to evaluate the statistical significance of differences.

## 3 Results

### 3.1 In Vitro Study Results

#### 3.1.1 Effect of 4MSK on Melanin Production

**FIGURE 1:**
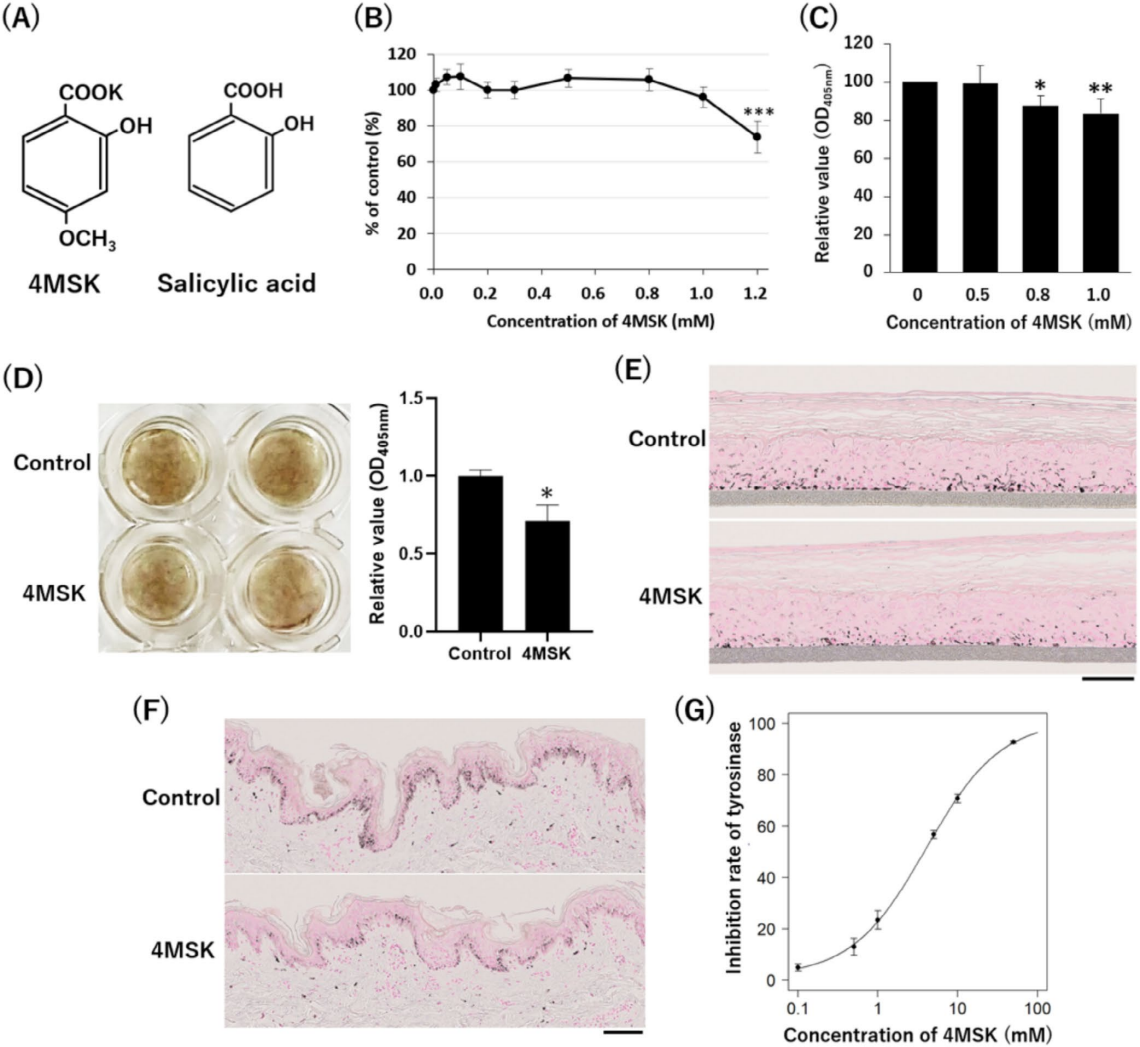
4MSK suppressed melanogenesis in human melanocytes. (A) The structure of potassium 4‐methoxysalicylate (left) and salicylic acid (right). (B) Viability of cultured normal human melanocytes treated with 0.01–1.2 mM 4MSK (*n* = 6). (C) Melanin content of human melanocytes treated with 0.5–1.0 mM 4MSK (*n* = 6). (D) Pigmentation of epidermal equivalents treated with or without 0.05% 4MSK. Amount of melanin in 3D models relative to that in the 0.05% 4MSK‐treated sample (*n* = 3). (E, F) Melanin content and localization in the epidermal equivalents (E) or in the ex vivo human skin explants (F) were visualized by Fontana‐Masson staining. (G) Inhibitory effect of 4MSK on the oxidation of L‐DOPA catalyzed by mushroom tyrosinase. Data are expressed as the mean ± SD of triplicate experiments. Scale bars; 100 μm. *p < 0.05, **p < 0.01, ***p < 0.005.

Structures of 4MSK and SA are shown in Figure 1A. Because of its structural similarity to SA, it was expected that 4MSK would exhibit inhibitory effects on melanogenesis similar to those established for SA [7, 8]. To ascertain whether 4MSK suppresses melanogenesis, we first investigated the effect of 4MSK on cultured normal human melanocytes. In the concentration range of 0.01–1.0 mM, 4MSK was not cytotoxic (Figure 1B). Melanin contents were significantly reduced in cells treated with 0.8 or 1.0 mM 4MSK compared with untreated cells (Figure 1C). Consistent with this result, melanin amounts in a human 3D epidermal equivalent were significantly reduced by treatment with 0.05% 4MSK (Figure 1D). The reduction of melanin contents in epidermal equivalents and ex vivo human skin explants was also confirmed by visualizing melanin with Fontana‐Masson staining (Figure 1E,F). Next, to investigate the mechanism by which 4MSK suppresses melanogenesis, the effect of 4MSK on tyrosinase activity was examined using mushroom tyrosinase. The results demonstrate that 4MSK inhibited tyrosinase activity in a concentration‐dependent manner (Figure 1G), as shown in the half‐maximal inhibitory concentration (IC_50_) curves in Figure 1G. The in vitro IC_50_ value of 4MSK was 4.02 mM.

#### 3.1.2 Effect of 4MSK on Differentiation of Keratinocytes

**FIGURE 2:**
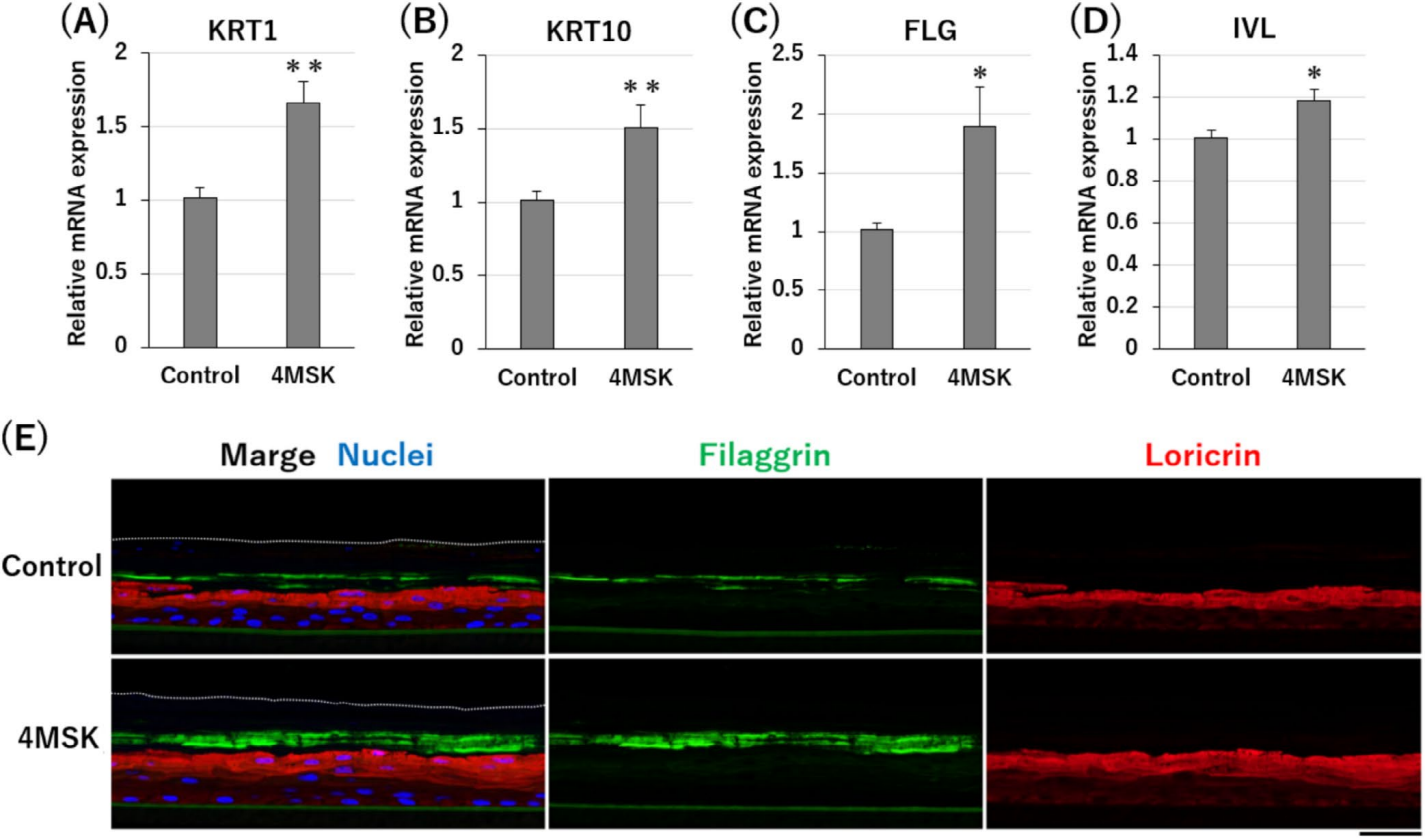
4MSK promoted gene expression of differentiation markers in keratinocytes. qPCR analysis of mRNA expression of KRT1 (A), KRT10 (B), FLG (C), and IVL (D) in 4MSK‐treated or untreated keratinocytes (mean ± SD, *n* = 8). *p < 0.05, **p < 0.01. (E) Epidermal equivalents were stained for filaggrin (green) and loricrin (red) protein expression analysis. Nuclei were stained with Hoechst 33342 (blue). Dotted lines represent the outermost layer of the epidermal equivalents. Scale bar; 50 μm.

Considering that SA has the effect of normalizing keratinocyte differentiation [9, 10], 4MSK is also expected to affect keratinocyte differentiation. To confirm this prediction, the gene expression of differentiation markers was examined with RT‐qPCR analysis. The gene expression of keratin 1 (*KRT1*) and keratin 10 (*KRT10*), two early differentiation markers of keratinocytes [11], was upregulated by treatment with 0.8 mM 4MSK for 2 days (Figure 2A,B). Moreover, the gene expression of filaggrin (*FLG*) and involucrin (*IVL*), two terminal differentiation markers [12, 13, 14], was also upregulated by 4MSK treatment (Figure 2C,D). To further confirm these findings, the expression of the terminal differentiation markers filaggrin and loricrin [15] was investigated in a human 3D epidermal equivalent model by immunohistochemistry. As shown in Figure 2E, the expression of filaggrin and loricrin was promoted in 4MSK‐treated skin models compared with untreated models.

### 3.2 Clinical Study Results

Thirty‐one Korean women aged 36–50 years (average: 43.5 years old) with facial hyperpigmentation were enrolled in this clinical study. During the clinical study period, there was no report or observation of skin reactions considered serious adverse events. Minor skin reactions such as itching (1/31), prickling (1/31), and burning sensation (1/31) were observed during the period (Table S2). These skin reactions were temporary and slight.

#### 3.2.1 Effect of 4MSK on Skin Lightness

**FIGURE 3:**
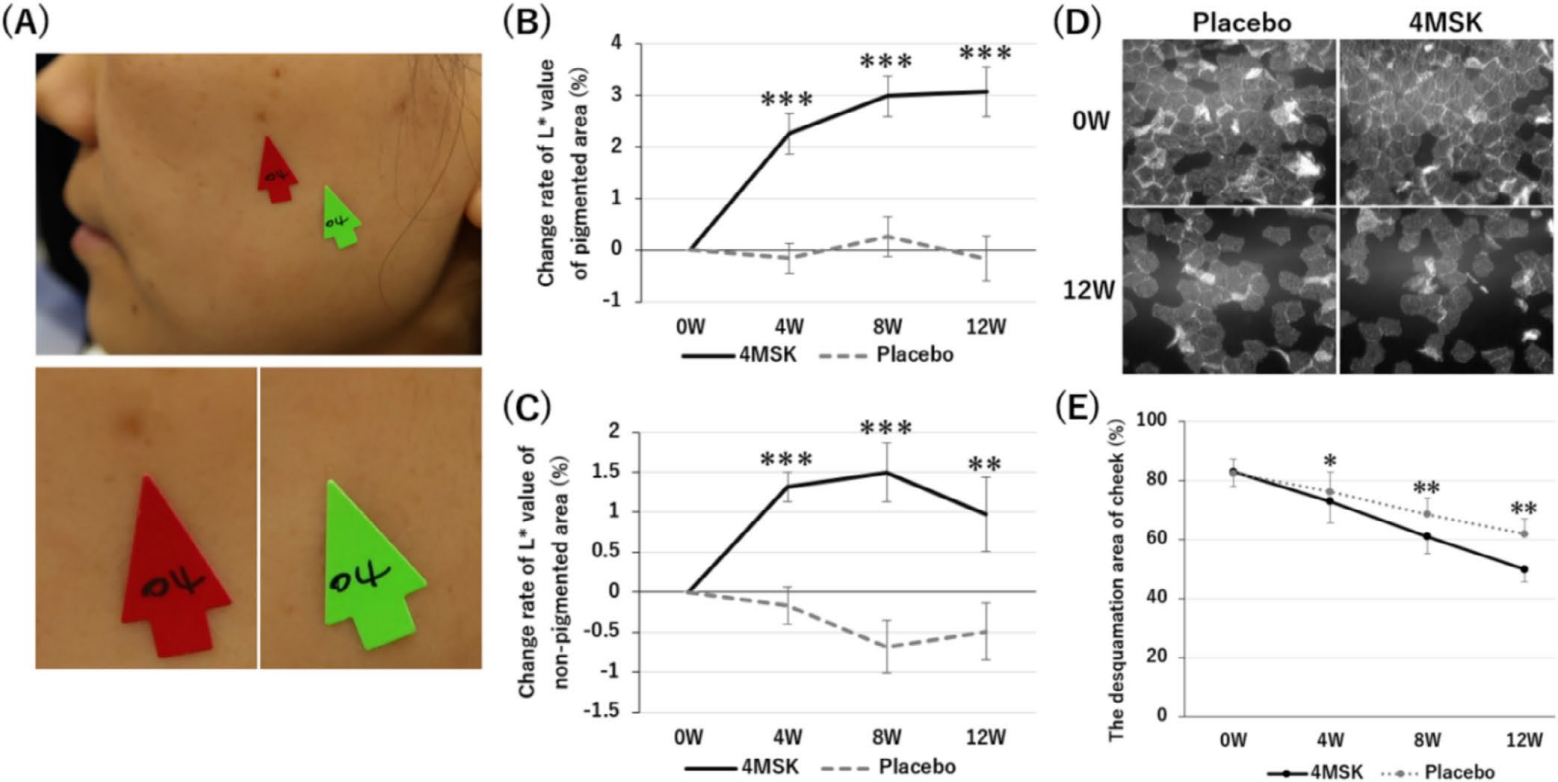
Efficacy of the 4MSK formulation in the human clinical study. (A) A representative image of the measurement site. The red arrow indicates a pigmented area (lower left), while the green arrow indicates a non‐pigmented area (lower right). (B) Change rate of L* value of the pigmented area. (C) Change rate of L* value of the non‐pigmented area. (D) Representative images of corneocytes collected by tape‐stripping. (E) The desquamation area ratio of the cheek. Data are expressed as the mean ± SD. *p < 0.05, **p < 0.01, ***p < 0.005 (vs. placebo). *n* = 31.

To investigate the effect of a 4MSK formulation on pigmented spots in vivo, a skin lightness parameter (L* value) of spots was measured using a spectrophotometer (Figure 3A, red arrow). After 4, 8, and 12 weeks of 4MSK treatment, the change rate of the L* value was significantly increased compared with baseline (Figure 3B, black line). In contrast, the placebo formulation did not increase the L* value from baseline (Figure 3B, gray dotted line). Furthermore, the change rate of the L* value of 4MSK‐treated spots was significantly higher after 4, 8, and 12 weeks of treatment compared with placebo‐treated spots (Figure 3B). Next, we ascertained the effect of 4MSK on non‐pigmented skin areas (Figure 3A, green arrow). After 4, 8, and 12 weeks of treatment, L* values of non‐pigmented areas applied with the 4MSK formulation were significantly higher compared with placebo‐treated areas (Figure 3C).

To further confirm the skin lightness effect of 4MSK, the VALUE parameter of a wide range of cheek areas was calculated from facial photographs taken with a VISIA skin analysis imaging system. The VALUE of 4MSK‐treated cheek areas was significantly higher compared with placebo‐treated areas after 4, 8, and 12 weeks of treatment (Figure S1A). Additionally, it was found that the deviation of the VALUE significantly decreased after 8 and 12 weeks of 4MSK treatment compared with placebo‐treated areas (Figure S1B).

#### 3.2.2 Effect of 4MSK on the Stratum Corneum

To investigate the effect of 4MSK on skin surface, the stratum corneum of cheeks was collected by tape stripping (Figure 3D). It was found that the desquamation areas of the cheek significantly decreased after 4, 8, and 12 weeks with either 4MSK or placebo treatment compared with baseline (Figure 3E). Furthermore, the desquamation area ratio of the 4MSK‐treated cheek was significantly lower than that of the placebo‐treated cheek (Figure 3E).

## 4 Discussion

The most common mechanism of action of skin lightening agents is the inhibition of the melanogenic process by inhibiting tyrosinase activity [3, 4]. Arbutin, 4‐*n*‐butylresorcinol (Rucinol), and ellagic acid inhibit tyrosinase activity [16, 17, 18, 19]. Similar to these active ingredients, 4MSK is commercially known to suppress melanin production by inhibiting tyrosinase activity [4]. However, the mechanism of action of 4MSK has not been described in scientific journals. Here, we confirmed that 4MSK reduced melanin contents in cultured human melanocytes, a human 3D epidermal equivalent model, and ex vivo human skin explants (Figure 1C–F). In addition, a tyrosinase assay showed that 4MSK inhibited the catalytic activity of tyrosinase in a concentration‐dependent manner, suggesting that one of the mechanisms by which 4MSK suppresses melanogenesis is through the direct inhibition of tyrosinase activity (Figure 1G). Liu et al. [5] suggests that SA downregulates the expression of tyrosinase, tyrosinase‐related protein 1 (*TYRP1*), tyrosinase‐related protein 2 (*TYRP2*), and microphthalmia‐related transcription factor (MITF), which is a master transcriptional regulator of these proteins in human melanocytes [7]. Because 4MSK has a similar chemical structure to SA (Figure 1A), it is also possible that 4MSK suppresses melanin production by controlling the gene expression of these melanogenesis‐related factors.

Keratinocytes migrate to the upper layer of the epidermis while differentiating and are finally excreted from the skin as dirt [20]. In the solar lentigo epidermis, expression of keratinocyte differentiation markers such as filaggrin and involucrin is downregulated and immature corneocytes are observed [21, 22]. Moreover, the number of stratum corneum cell layers is reportedly increased in sun‐exposed skin, suggesting a reduced keratinization rate and delayed epidermal turnover [22]. These findings indicate that melanin excretion via epidermal turnover is stagnant in pigmented skin areas. In this study, we found that 4MSK upregulated both early and terminal differentiation markers in human keratinocytes (Figure 2), suggesting that 4MSK can improve delayed keratinization rates and melanin excretion in pigmented skin areas.

**FIGURE 4:**
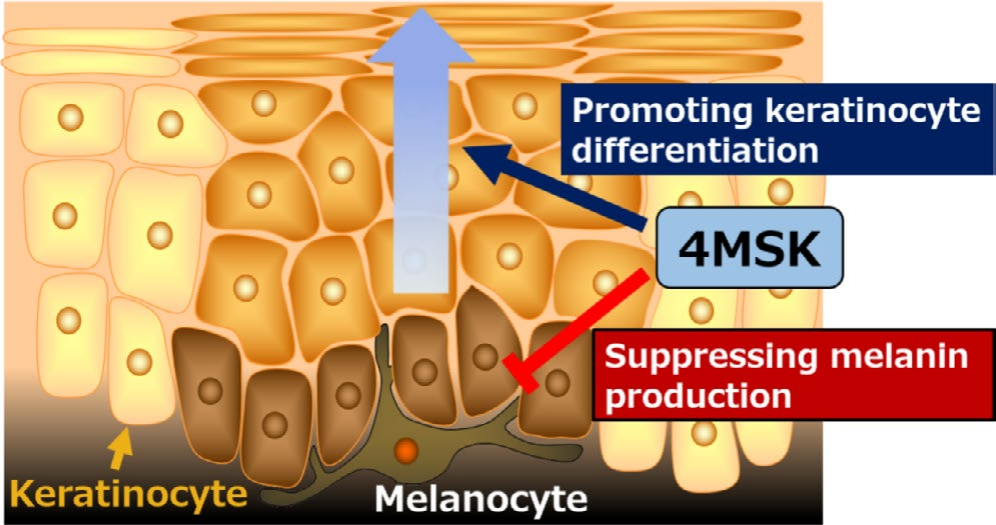
Model of the mechanisms by which 4MSK reduces skin pigmentation. 4MSK suppresses melanogenesis by inhibiting tyrosinase activity. In addition, 4MSK possibly accelerates epidermal turnover by promoting keratinization. Both mechanisms contribute to the suppression and reduction of skin pigmentation.

Our in vitro experiments demonstrate that 4MSK acts on both melanocytes and keratinocytes to reduce skin pigmentation (Figure 4). However, the effectiveness of 4MSK against hyperpigmentation has not been reported in vivo. We investigated whether a 4MSK formulation enhanced skin lightness values of facial pigmentation in a human study (Figure 3). Remarkably, the 4MSK formulation not only reduced pigmented skin areas, but it enhanced lightness in the overall cheek area, including non‐pigmented sites (Figure 3C and Figure S1A). Because 4MSK is suggested to reduce the variation in skin lightness values in the cheek area (Figure S1B), it is considered to uniformly increase skin lightness, rather than locally as in leukoderma. Indeed, there was no serious skin reaction such as leukoderma during the course of this clinical study (Table S2). In addition, it was reported that 4MSK is not a leukoderma‐inducing phenol that is oxidized by tyrosinase to form *ortho*‐quinones [23]. Taken together with the current results, 4MSK is expected to safely reduce pigmentation and uneven lightness of skin. Further safety evaluations, including in vitro and ex vivo experiments, will clarify its safety.

Our unpublished data suggest that 4MSK does not have a strong peeling effect as SA. However, it was unclear how long‐term continuous application of 4MSK would affect the skin surface. Our clinical study showed that continuous application of a 4MSK formulation reduced the desquamation area ratio (Figure 3E), suggesting that 4MSK has a mild peeling effect that contributes to the exfoliation of old stratum corneum from the skin surface. The underlying mechanisms of the mild peeling effect remain unclear but might be due to the dissolving effect of desmosomal proteins, as suggested for that of SA [24]. Considering these results together with its promoting effect on keratinocyte differentiation, 4MSK is expected to accelerate epidermal turnover.

Collectively, our findings demonstrate that 4MSK reduces skin pigmentation and evenly brightens the skin by suppressing melanogenesis and promoting keratinocyte differentiation.

## Author Contributions

Y.S. wrote the manuscript; Y.S., T.S., and K.H. conceived and designed the project, and analyzed all the data; S.K. and M.H. performed the experiments on melanogenesis; T.S., M.I., and A.S. conceived and designed the clinical study; Y.F.: discussed and reviewed all the data.

## Ethics Statement

This study was approved by the ethics committees of both Shiseido Co., Ltd. (IRB number: B01620) and Institut d'Expertise (IRB number: IECK(2)‐IRB‐19K516452) and conducted under the Ethical Guidelines for Clinical Research and the Declaration of Helsinki (UMIN000029305).

## Consent

All participants received an adequate explanation of the clinical study and provided written informed consent.

## Conflicts of Interest

Authors Y.S., T.S., S.K., M.H., K.H., M.I., and A.S. are employees of Shiseido Co., Ltd.

## Supporting information


Data S1.

## Data Availability

The data that support the findings of this study are available from the corresponding author upon reasonable request.
