# Correction to “Potassium 4‐methoxysalicylate (4MSK) exerts a skin lightening effect by acting on melanocytes and keratinocytes”

**DOI:** 10.1111/jocd.70317

**Published:** 2025-06-30

1

Shirasugi Y, Shibata T, Koike S, Hara M, Hasegawa K, Iino M, Sonoki A, Funasaka Y. *J Cosmet Dermatol*. 2025 Mar;24(3):e70112.

In paragraph 7 of the “Materials and Methods” section, the UMIN ID provided is incorrect, and our clinical study has not been registered in any registries approved by ICMJE.

We apologize for this error.

