# Supplementary material for: Potassium 4‐Methoxysalicylate (4MSK) Exerts a Skin Lightening Effect by Acting on Melanocytes and Keratinocytes
Source: J Cosmet Dermatol. 2025 Mar 12;24(3):e70112. doi: 10.1111/jocd.70112 (PMC11898116; doi:10.1111/jocd.70112)
Supplement: Supplementary file 1 — Data S1. [file JOCD-24-e70112-s001.docx]

**Supplementary Materials**

Supplemental Table 1. Primers for quantitative polymerase chain reaction.

Supplemental Table 2. Skin reactions during the clinical study.


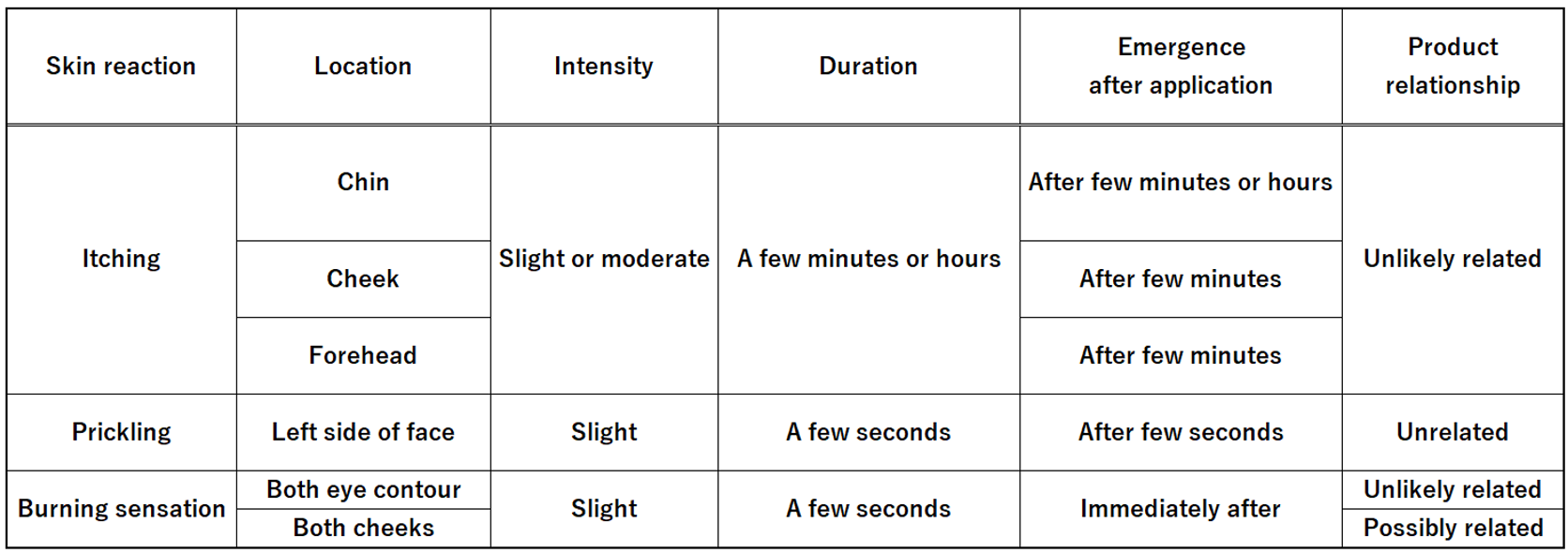


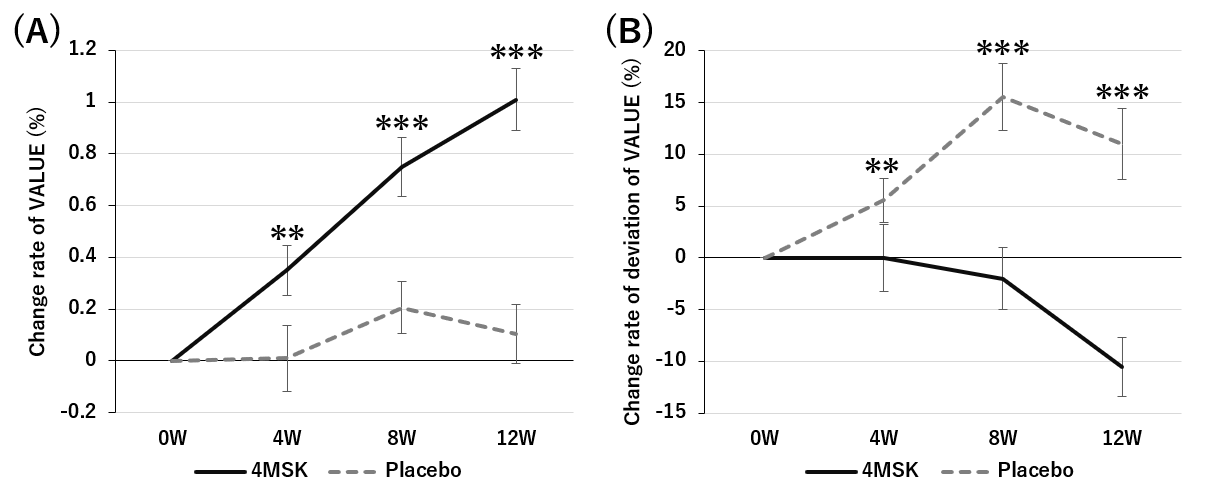


Supplemental Figure 1. The 4MSK formulation increased VALUE parameters of the cheek area.

(A) Change rate of VALUE of the cheek area. (B) Change rate of the deviation of VALUE for each subject. Data are expressed as the mean ± SD. **p < 0.01, ***p < 0.005 (vs placebo). n = 31.
